# Extrauterine growth restriction in preterm infants: Postnatal growth pattern and physical development outcomes at age 3–6 years

**DOI:** 10.3389/fped.2022.945422

**Published:** 2022-07-29

**Authors:** Siyuan Lan, Huanhuan Fu, Rui Zhang, Guimei Zhong, Liya Pan, Fei Bei, Li Hong

**Affiliations:** ^1^Department of Clinical Nutrition, Shanghai Children’s Medical Center, School of Medicine, Shanghai Jiao Tong University, Shanghai, China; ^2^Department of Pediatrics, Fujian Provincial Maternity and Children’s Hospital, Fujian Medical University, Fujian, China; ^3^Department of Neonatal Intensive Care Unit, Quanzhou First Hospital, Fujian, China; ^4^Department of Neonatology, Shanghai Children’s Medical Center, School of Medicine, Shanghai Jiao Tong University, Shanghai, China

**Keywords:** extrauterine growth restriction, preterm infants, growth pattern, obesity, short stature, thinness

## Abstract

**Objectives:**

To investigate the postnatal growth trajectories of preterm infants and evaluate the association between extrauterine growth restriction (EUGR) at discharge and adverse physical growth outcomes at age 3–6 years.

**Methods:**

Premature infants admitted to Shanghai Children’s Medical Center within 24 h after birth from 1 January 2016 to 31 December 2018 were enrolled. Neonatal complications, nutrition support, and anthropometric data were collected and analyzed to diagnose EUGR on different definitions at discharge. The weight and the height of each subject were collected by telephone investigation from 1 September 2021 to 31 November 2021 to access the incidences of overweight/obesity, short stature, and thinness at age 3–6 years.

**Results:**

A total of 527 preterm infants were included in the final sample. The overall mean weight and height *Z*-scores were –0.37 ± 0.97 SD and –0.29 ± 1.18 SD at birth, and increased to –0.03 ± 1.11 SD and 0.13 ± 1.2 SD at follow-up, respectively. The logistic regression analysis indicated longitudinal EUGR on head circumference as the risk factor of overweight or obesity, cross-sectional EUGR on height as the risk factor of short stature, and delayed EN as the risk factor of thinness.

**Conclusion:**

The growth trajectories of the preterm newborns tended toward the normal direction. Longitudinal EUGR on the head circumference and cross-sectional EUGR on height at discharge were associated with adverse physical growth outcomes at age 3–6 years.

## Introduction

Extrauterine growth restriction (EUGR) refers to preterm growth failure which an anthropometric measure is below the standard based on the postmenstrual age (gestational age + days of hospitalization) (1). As a result of prenatal conditions, insufficient nutritional intakes and a range of mild-to-severe complications, EUGR potentially caused the impairment on the physical growth and neurodevelopment in both acute and delayed forms (2, 3).

So far, there is no unanimity on the diagnosis of EUGR. The incidence of EUGR varies from 13to 97% because of the inconsistent definitions used (1). The cross-sectional EUGR is identified as weight, length, or head circumference (HC) below the 10th percentile based on the postmenstrual age, while the longitudinal EUGR is defined as the decrease in weight, length, or HC *Z*−scores>1 SD between birth and the assessment time (4). De Rose et al. (5) found that the longitudinal EUGR was more predictable in neurodevelopmental outcomes at a 2−year follow-up compared to the cross-sectional EUGR. A follow-up survey of 103 children with severe cross-sectional EUGR (*Z*−scores in weight or length less than −2 SD at discharge) showed that at an average age of 3.9 ± 1.7 years, children with *Z*−scores in height, weight, and body mass index (BMI) below −2 SD still accounted for 12.6, 13.6, and 18.4% of the study population, respectively (6).

Additionally, preterm infants with EUGR are at higher risk of metabolic disorders triggered by the growth failure and subsequent catch-up growth (7). A review done by Ong et al. (8) indicated that the rapid weight gain was associated with insulin resistance and cardiovascular disease in several observational studies. Similarly, a meta-analysis of 47,661 individuals from 10 cohort studies reported the correlation between the accelerated postnatal growth and obesity in later life (9). However, the direct relationship between EUGR and obesity has not been demonstrated yet.

The objectives of this study were to investigate the postnatal growth trajectories of preterm infants, and to evaluate whether the influencing factors of EUGR and the diagnosis of EUGR at discharge have effects on the adverse physical growth outcomes including overweight/obesity, short stature, and thinness at 3–6 years of follow-up.

## Materials and methods

### Subjects

The retrospective, single-center study was conducted from 1 January 2016 to 31 December 2018 at a neonatal intensive care unit of Shanghai Children’s Medical Center affiliated to Shanghai Jiao Tong University School of Medicine. The criteria for enrollment were as follows: (i) Gestation age was less than 37 weeks, (ii) admission in 24 h after birth, and (iii) the length of the stay was more than or 7 days. The exclusion criteria were as follows: (i) Birth with major congenital anomalies, (ii) major surgery during hospitalization, (iii) death or discharge against medical advice, and (iv) incomplete medical records. The participants who were unable to contact or provide the precise information were regarded as loss to follow-up. A total of 527 subjects were included in the final sample and divided into group 2016 (*n* = 169), group 2017 (*n* = 160), and group 2018 (*n* = 198) based on the birth year, with an average age of 5.35 ± 0.26 years, 4.34 ± 0.31 years, and 3.26 ± 0.28 years at follow-up, respectively. According to the growth material at follow-up, 79 children were classified as overweight/obesity, 20 as short stature, and 35 as thinness. Written informed consent was obtained from subjects’ parents. Approval from the Ethics Committee of Shanghai Children’s Medical Center was obtained. All methods were performed in accordance with the relevant guidelines and regulations.

### Data collection

Body measurements, clinical characteristics, and nutrition intakes during hospitalization were extracted from the electronic medical charts of each subject. Weight was measured without clothes daily by an electronic scale before feeding, and after changing diapers. Height and HC were measured by trained nurses weekly with non-stretch measuring tapes. Growth *Z*−scores and percentiles were calculated based on the postmenstrual age by Fenton 2013 growth charts (10). The clinical characteristics including gestational age, gender, multiple births, hypertensive disorders of pregnancy (HDP), gestational diabetes mellitus (GDM), neonatal asphyxia, invasive ventilation, bronchopulmonary dysplasia (BPD), grade III−IV of intracranial hemorrhage (ICH), neonatal sepsis, and necrotizing enterocolitis (NEC) were recorded. For enteral nutrition (EN), data on the type (preterm formula milk or breast milk) and amount of enteral feeding, day of initiation, and days to achieve exclusive EN were collected. For parenteral nutrition (PN), information on the day of initiation and termination, duration, and the energy intakes during the first week after birth, on the first day after PN termination, and at discharge were included. Weight (cm) and height (kg) at the follow-up of each subject were collected by telephone investigation from 1 September 2021 to 31 November 2021. The *Z*-scores and the percentiles of height, weight, and BMI at follow-up were calculated according to the World Health Organization (WHO) growth curve.

## Nutrition strategy

The nutritional schedules of all subjects were personalized by neonatologists and nutritionists depending on the physiological and pathological conditions. Enteral nutrition was initiated mostly at day 2 after birth, unless postponed or stopped by (i) digestive tract obstruction caused by congenital gastrointestinal malformation, (ii) suspected or diagnostic NEC, (iii) hemodynamic instability, and (iv) multiple organ dysfunction. Breast milk feeding was considered as the first choice for subjects and preterm formula milk was available to offset the lack or deficiency of breast milk. Inadequate EN intakes and EN calories more than or 80 cal/kg per day, respectively, were the signs of the initiation and discontinuation of PN, which was prescribed by nutritionists based on the Chinese guidelines for nutrition support in neonates from 2016 to 2017 (11) and adjusted by the latest version in 2018 (12).

### Definitions

Small for gestational age (SGA), appropriate for gestational age (AGA), and large for gestational age (LGA) are defined as the birth weight less than 10th percentile, between 10th and 90th percentiles, and above 90th percentile, respectively, according to the Fenton 2013 growth chart (10). The cross−sectional EUGR on weight, height, and HC are diagnosed as weight-for-age *Z*−score (WAZ), height−for−age *Z*−score (HAZ), and HC−for−age *Z*−score (HCAZ) less than −1 SD at discharge, and longitudinal EUGR on weight, height, and HC are diagnosed as the decline of WAZ, HAZ, and HCAZ above 1 SD from birth to discharge, respectively (4). At 3−6 years of follow-up, BMI-for-age above 85th percentile was identified as overweight/obesity, HAZ below −2 SD as short stature, and BMI−for−age *Z*−score (BMIZ) below −2 SD as thinness based on the WHO growth curve (13). In this study, human milk intakes reaching more than half the amount of total enteral feeding is considered breast milk feeding.

### Statistical analysis

The categorical variables were given as numbers (percentages) and compared using the Chi−squared or Fisher’s exact test. The normal distributed quantitative variables were expressed as mean ± SD and compared using the Student’s *t*-test or one-way ANOVA test. The non−normal distributed quantitative variables were expressed as the median (interquartile range) and compared using the Mann–Whitney U test or Kruskal−Wallis test. Comparison between the two of the three groups were performed by Bonferroni’s adjustments or Mann–Whitney U test. The contributing factors for the body-build of preterm infants at 3–6 years of age were estimated using a multivariate logistic regression (method: forward: LR). The factors with significant differences in group comparison were included as independent variables. Birth weight, longitudinal EUGR for HC, weight at discharge, changes in HCAZ during hospitalization, and changes in WAZ during follow-up were entered into the overweight/obesity model. Birth weight, SGA, cross-sectional EUGR for weight, height and HC, and changes in WAZ and HAZ during follow-up were entered into the short stature model. Days to start EN, and changes in WAZ and HAZ during follow-up were entered into the thinness model. The statistical significance was set at *p* < 0.05. All statistical data were analyzed using the SPSS statistical software package, version 22.0.

## Results

**FIGURE 1:**
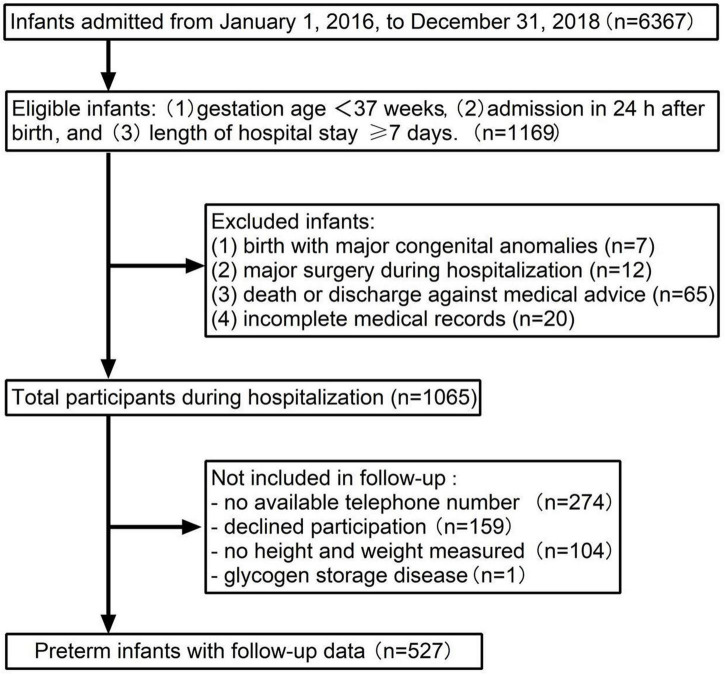
Flowchart of participants.

Inclusion and exclusion criteria of participants are presented in Figure 1. A total of 6,367 newborns admitted to Shanghai Children’s Medical Center from 1 January 2016 to 31 December 2018, and 1,169 preterm infants met the criteria for the enrollment. A total of 104 newborns with congenital anomalies at birth, major surgery during hospitalization, incomplete medical records, and death or discharge against medical advice were excluded. There were 1,065 preterm infants eligible for the follow-up investigation. A total of 274 patients with no available telephone number, 159 with declined participation, 105 with no weight or height measured, and 1 case with glycogen storage disease were not included in the final sample.

**FIGURE 2:**
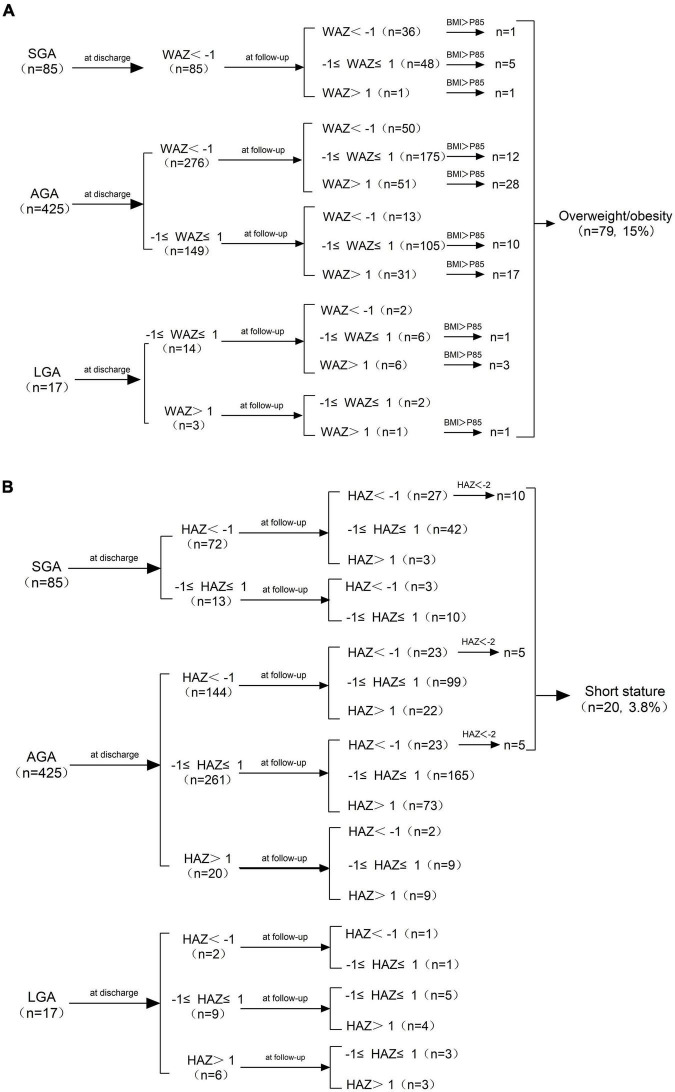
**(A)** Changes in WAZ from birth to age 3−6 years and the distribution of overweight/obesity at follow-up. **(B)** Changes in HAZ from birth to age 3−6 years and the distribution of short stature at follow-up. *SGA, small for gestational age; AGA, appropriate for gestational age; LGA, large for gestational age; WAZ, weight-for-age Z-score; HAZ, height−for−age Z−score; BMI, Body Mass Index.*

As shown in Figure 2A, the WAZ of all the SGA infants were lower than -1 SD at discharge, while 57.6% (49/85) of them achieved weight catch-up growth. The incidence of cross-sectional EUGR on weight at discharge in AGA infants was 64.9% (276/425), and 78.2% (216/276) of these infants recovered (WAZ ≥ −1 SD) at follow-up. The WAZ of all the LGA infants were larger than −1 SD at discharge, and 29.4% (5/17) of them were overweight or obese at follow-up. As shown in Figure 2B, the HAZ of 27 SGA infants were below −1 SD at discharge, of which 11.76% (10/27) were short-stature at follow-up. The risk of short stature in AGA infants was 2.4% (10/425), of which 5 had a HAZ less than −1 SD and 5 had a HAZ between −1 SD and 1 SD at discharge. None of the 17 LGA infants developed short stature at follow−up.

**FIGURE 3:**
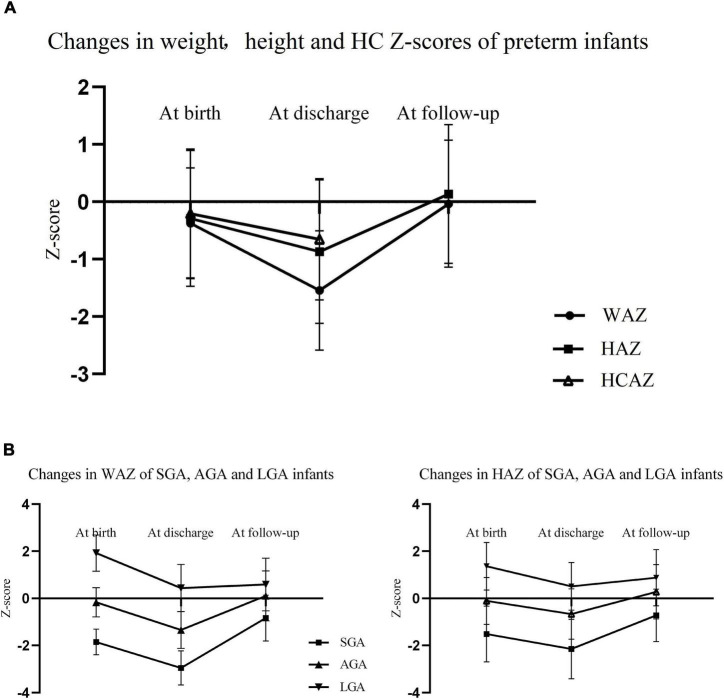
**(A)** Changes in WAZ, HAZ, and HCAZ from birth to age 3–6 years of preterm infants (the measurement of HC was not applicable for children at age 3−6 years); **(B)** changes in WAZ and HAZ from birth to age 3−6 years of SGA, AGA, and LGA preterm infants. *WAZ, weight-for-age Z−score; HAZ, height-for-age Z−score; HC, head circumference*; *HCAZ HC-for-age Z−score; SGA, small for gestational age; AGA, appropriate for gestational age; LGA, large for gestational age.*

Changes in weight, height, and HC *Z*−scores in preterm infants are exhibited in Figure 3. There was a noticeable decrease in *Z*−scores for weight, height, and HC from birth to discharge, and WAZ declined more sharply than HAZ and HCAZ. Both of WAZ and HAZ rebounded significantly and came close to zero at follow−up as shown in Figure 3A. As depicted in Figure 3B, SGA infants caught up with their peers while LGA infants lost their advantages at birth and were presented with decrease in both WAZ and HAZ after discharge.

The general characteristics of preterm infants are presented in Table 1. A total of 527 preterm infants were divided into group 2016 (*n* = 169), group 2017 (*n* = 160), and group 2018 (*n* = 198) based on the admission time. Gestational age in group 2017 was significantly larger and the decrease in WAZ during hospitalization was less than other groups. In group 2018, more preterm newborns with lower birth weight and of multiple births were admitted and experienced longer hospital stays. There was no discrepancy among all groups in WAZ, HAZ, and HCAZ at birth, those at discharge, changes in WAZ and HAZ during follow-up, and the occurrence of short stature and overweight/obesity at follow-up among these groups.

**TABLE 1:** The general characteristics of preterm infants before and after discharge.

Characteristics	Total number(*n* = 527)	2016 (*n* = 169)	2017 (*n* = 160)	2018 (*n* = 198)	*p*
Gestation age (weeks)	33.43(31.43,34.57)	32.29(30.43,34)	34(33,34.96)	33.29(31,34.57)	<0.001 *^ab^*
Male (%)	288(54.6)	89(52.7)	98(61.3)	101(51)	0.126
Multiple births (%)	195(37)	60(35.5)	49(30.6)	86(43.4)	0.039 *^b^*
Birth weight (g)	1875(1530,2155)	1900(1605,2137.5)	1990(1552.5,2208.8)	1747.5(1366.25,2082.5)	0.003^bc^
WAZ at birth (SD)	−0.3(−1.03,0.23)	−0.35(−1.06,0.27)	−0.26(−1.02,0.23)	−0.29(−0.95,0.23)	0.975
HAZ at birth (SD)	−0.21(−1.08,0.51)	−0.29(−1.05,0.47)	−0.24(−1.23,0.32)	−0.14(−0.96,0.65)	0.182
HCAZ at birth (SD)	−0.16(−0.84,0.57)	−0.18(−0.84,0.51)	−0.17(−0.98,0.5)	−0.11(−0.76,0.59)	0.253
Length of hospital stay (days)	21.5(14.5,34)	19.5(14.25,28.5)	20(13.5,30.5)	25.5(15,41.63)	0.003^bc^
Age at discharge (weeks)	36.71(35.86,38)	36.71(35.71,37.86)	36.71(36,37.86)	36.86(36.14,38.14)	0.144
Weight at discharge (g)	2150(2030,2370)	2115(1990,2342.5)	2200(2045,2418.8)	2145(2020,2325)	0.013 *^a^*
WAZ at discharge (SD)	−1.43(−2.19,−0.85)	−1.5(−2.2,−0.81)	−1.29(−2.01,−0.83)	-1.48(−2.33,−0.95)	0.188
HAZ at discharge (SD)	−0.75(−1.61,−0.05)	−0.77(−1.61,−0.17)	−0.59(−1.62,0.14)	−0.83(−1.62,−0.14)	0.189
HCAZ at discharge (SD)	−0.56(−1.32,0.03)	-0.56(-1.46,0.06)	-0.58(-1.38,-0.03)	−0.51(−1.18,0.03)	0.584
Changes in WAZ during hospitalization	−1.08(−1.35,−0.83)	−1.1(−1.28,−0.86)	−1.02(−1.32,−0.75)	−1.11(−1.46,−0.84)	0.041 *^b^*
Changes in HAZ during hospitalization	−0.55(−1.13,−0.04)	−0.62(−1.16,−0.15)	−0.33(−0.86,0.22)	−0.62(−1.28,−0.21)	<0.001 *^ab^*
Changes in HCAZ during hospitalization	−0.42(−0.99,0.19)	−0.46(−1,0.16)	−0.41(−0.96,0.21)	−0.42(−1,0.19)	0.772
Age at follow-up (years)	4.25(3.41,5.12)	5.36(5.14,5.55)	4.34(4.09,4.58)	3.28(3.06,3.46)	<0.001 *^abc^*
WAZ at follow-up (SD)	−0.03 ± 1.11	−0.01 ± 1.1	0.13 ± 1.07	−0.19 ± 1.13	0.025 *^b^*
HAZ at follow-up (SD)	0.12(−0.61,0.89)	0.09(−0.62,1.02)	0.2(−0.64,0.78)	0.09(−0.58,0.82)	0.697
BMIZ at follow-up (SD)	−0.27(−0.97,0.46)	−0.21(−0.97,0.45)	−0.13(−0.67,0.74)	−0.46(−1.14,0.26)	0.002 *^b^*
Changes in WAZ during follow-up (SD)	1.51 ± 1.23	1.54 ± 1.38	1.56 ± 1.1	1.45 ± 1.2	0.626
Changes in HAZ during follow-up (SD)	0.99(−0.01,1.86)	1.12(0.05,2.02)	0.76(−0.04,1.75)	1.04(−0.06,1.87)	0.251
WAZ<-1SD at follow-up (%)	101(19.2)	35(20.7)	22(13.8)	44(22.2)	0.106
HAZ<-1SD at follow-up (%)	79(15)	24(14.2)	27(16.9)	28(14.1)	0.726
WAZ<-2SD at follow-up (%)	25(4.7)	5(3)	6(3.8)	14(7.1)	0.141
HAZ<-2SD at follow-up (%)	20(3.8)	6(3.6)	8(5)	6(3)	0.612
Thinness at follow-up (%)	35(6.6)	19(11.2)	2(1.3)	14(7.1)	<0.001 *^ab^*
Overweight/obesity at follow-up (%)	79(15)	27(16)	32(20)	20(10.1)	0.12

a, a significant difference between groups 2016 and 2017; b, a significant difference between groups 2017 and 2018; c, a significant difference between groups 2016 and 2018. WAZ, weight-for−age Z-score; HAZ, height-for-age Z−score; HC, head circumference; HCAZ, HC−for−age Z-score; BMI, Body Mass Index; BMIZ, BMI-for-age Z−score.

Table 2 shows the neonatal complications and nutrition support of preterm infants. Significantly higher rates of HDP and lower rates of invasive ventilation and BPD were observed in group 2018 than that in group 2017, while there was no difference in the morbidity of SGA, GMD, neonatal asphyxia, grades III−IV of ICH, neonatal sepsis, and NEC. It took fewer days to start EN and PN for newborns in group 2018, and there were no differences in the duration of PN and days to reach full feeds. The maximum energy intakes during the first week after birth and energy intakes at discharge were increasing every year. There was no significant difference in the diagnosis of EUGR observed, except longitudinal EUGR on height at discharge.

**TABLE 2:** The neonatal complications and nutritional support of preterm infants during hospitalization.

Characteristics	Total number (*n* = 527)	2016 (*n* = 169)	2017 (*n* = 160)	2018 (*n* = 198)	*P*-value
SGA (%)	85(16.1)	28(16.6)	25(15.6)	32(16.2)	0.973
HDP (%)	131(24.9)	42(24.9)	50(31.3)	39(19.7)	0.042 *^b^*
GDM (%)	82(15.6)	26(15.4)	23(14.4)	33(16.7)	0.835
Invasive ventilation (%)	180(34.2)	48(28.4)	45(28.1)	87(43.9)	0.001 *^b^*
BPD (%)	88(16.7)	15(8.9)	18(11.3)	55(27.8)	<0.001 *^b^*
Grades III–IV of ICH (%)	9(1.7)	5(3)	2(1.3)	2(1)	0.335
Neonatal sepsis (%)	9(1.7)	3(1.8)	1(0.6)	5(2.5)	0.339
NEC (%)	12(2.3)	4(2.4)	3(1.9)	5(2.5)	0.913
Breast milk feeding(%)	87(16.5)	20(11.8)	23(14.4)	44(22.2)	0.019 *^c^*
Days to start EN (days)	2(2,3)	2(2,3.75)	2(2,3)	2(2,3)	0.016^c^
Days to start PN (days)	3(2,4)	3(2,4)	3(2,4)	2(2,3)	<0.001 *^bc^*
Duration of PN (days)	14.5(9,22)	15(10,22.25)	12(8,23)	15(9,22)	0.252
Days to reach full feeds (days)	17(12,25)	17(12,24)	15(12,25)	18(12.5,26)	0.507
Days to regain birth weight (days)	11(8,13)	11(9,14)	10(8,12)	11(8,13)	0.021 *^a^*
Maximum energy intakes during the first week after birth (kcal/kg/day)	91.2(82.7,99.6)	91.2(84,97.7)	88(79.7,96.1)	93.6(85.6,102.7)	0.003^b^
Energy intakes on the first day after PN termination (kcal/kg/day)	84.5(80.8,90.2)	83.9(80.7,89.8)	83.7(80,90)	85.6(81.5,90.6)	0.151
Energy intakes at discharge (kcal/kg/day)	111.2(100.3,119)	104.4(95.8,114.8)	111.2(101.3,118.1)	115.6(105.6,122.4)	<0.001 *^abc^*
Cross-sectional EUGR for weight at discharge (%)	361(68.5)	114(67.5)	104(65)	143(72.2)	0.332
Cross-sectional EUGR for height at discharge (%)	218(41.4)	70(41.4)	64(40)	84(42.4)	0.898
Cross-sectional EUGR for HC at discharge (%)	179(34)	59(34.9)	58(36.3)	62(31.3)	0.588
Longitudinal EUGR for weight at discharge (%)	310(58.8)	108(63.9)	83(51.9)	119(60.1)	0.077
Longitudinal EUGR for height at discharge (%)	155(29.4)	54(32)	28(17.5)	73(36.9)	<0.001^ab^
Longitudinal EUGR for HC at discharge (%)	130(24.7)	43(25.4)	38(23.8)	49(24.7)	0.938

a, a significant difference between groups 2016 and 2017; b, a significant difference between groups 2017 and 2018; c, a significant difference between groups 2016 and 2018. SGA, small for gestational age; HDP, hypertensive disorders of pregnancy; GDM, gestational diabetes mellitus. BPD, bronchopulmonary dysplasia; ICH, intracranial hemorrhage; NEC, necrotizing enterocolitis; EN, enteral nutrition; PN, parenteral nutrition; EUGR, extrauterine growth restriction; HC, head circumference.

The characteristics of preterm infants with overweight/obesity, short stature, and thinness at age 3−6 years are presented in Table 3. The children with overweight/obesity had significantly higher weight at birth and at discharge, and greater decrease in WAZ during hospitalization. The children with short stature had remarkably lower weight at birth and at discharge, lower energy intakes at discharge, a higher rate of SGA, and longer length of hospitalization. The children with thinness started EN later and had greater decrease in HAZ during hospitalization. The diagnosis of longitudinal EUGR on HC at discharge was associated with overweight/obesity, while the diagnosis of cross-sectional EUGR on weight, height, and HC at discharge was related to short stature at follow-up.

**TABLE 3:** Comparisons of preterm infants with or without overweight/obesity, short stature, and thinness at age 3–6 years.

Characteristics	Overweight/obesity (BMI > P85, *n* = 79)	BMI ≤ P85 (*n* = 448)	Short stature (HAZ < -2, *n* = 20)	HAZ ≥ -2 (*n* = 507)	Thinness (BMIZ < -2, *n* = 35)	BMIZ ≥ −2 (*n* = 492)
Gestation age (w)	33.29(32,34.57)	33.43(31.43,34.57)	33.21(32.25,33.96)	33.43(31.43,34.57)	32.57(30.43,34.14)	33.43(31.57,34.68)
Birth weight (g)	1986.2 ± 544.8*^a^*	1838.2 ± 488.3*^a^*	1555(1125,1746)*^b^*	1890(1540,2180)*^b^*	1776.9 ± 483.5	1866.3 ± 500.5
Male (%)	47(59.5)	241(53.8)	9(45)	279(55)	18(51.4)	270(54.9)
Multiple births (%)	27(34.2)	168(37.5)	8(40)	187(36.9)	18(51.4)	177(36)
SGA (%)	7(8.9)	78(17.4)	10(50)*^b^*	10(2)*^b^*	8(22.9)	77(15.7)
HDP (%)	17(21.5)	14(3.1)	5(25)	126(24.9)	10(28.6)	121(24.6)
GDM (%)	15(19)	67(15)	0(0)	82(16.2)	2(5.7)	80(16.3)
Neonatal asphyxia (%)	9(11.4)	73(16.3)	5(25)	77(15.2)	9(25.7)	73(14.8)
Invasive ventilation (%)	31(39.2)	149(33.3)	6(30)	174(34.3)	12(34.3)	168(34.1)
BPD (%)	13(16.5)	75(16.7)	3(15)	85(16.8)	8(22.9)	80(16.3)
Grade III-IV of ICH (%)	1(1.3)	8(1.8)	1(5)	8(1.6)	2(5.7)	7(1.4)
Neonatal sepsis (%)	0(0)	9(2)	0(0)	9(1.8)	0(0)	9(1.8)
NEC (%)	2(200)	10(2.2)	2(10)	10(2)	1(2.9)	11(2.2)
Breast milk feeding(%)	15(19)	72(16.1)	2(10)	85(16.8)	7(20)	80(16.3)
Cross-sectional EUGR for weight (%)	47(59.5)	314(70.1)	19(95)*^b^*	342(67.5)*^b^*	26(74.3)	335(68.1)
Cross-sectional EUGR for height (%)	28(35.4)	190(42.4)	15(75)*^b^*	203(40)*^b^*	20(57.1)	198(40.2)
Cross-sectional EUGR for HC (%)	24(30.4)	155(34.6)	13(65)*^b^*	166(32.7)*^b^*	16(45.7)	163(33.1)
Longitudinal EUGR for weight (%)	44(55.7)	266(59.4)	12(60)	298(58.8)	21(60)	289(58.7)
Longitudinal EUGR for Height (%)	23(29.1)	132(29.5)	5(25)	150(29.6)	15(42.9)	140(28.5)
Longitudinal EUGR for HC (%)	28(35.4)*^a^*	102(22.8)*^a^*	1(5)	129(25.4)	12(34.3)	118(24)
Days to start EN (d)	2(2,3)	2(2,3)	2(2,4)	2(2,3)	3(2,4)*^c^*	2(2,3)*^c^*
Days to start PN (d)	3(2,4)	3(2,4)	2(2,4.25)	3(2,4)	3(2,4)	3(2,4)
Duration of PN (d)	15(11,28.5)	14(9,22)	16(10.75,25.75)	14(9,22)	17(12,25.75)	14(9,22)
Days to reach full feeds (d)	16(12,25.5)	17(12,25)	22(15.25,27.75)	17(12,25)	20(14,26)	17(12,25)
Days to regain birth weight (d)	11(9,13)	11(8,13)	9(7.25,11.75)	11(9,13)	11.5(8.25,13)	11(8,13)
Energy intakes on the first day after PN termination (Kcal/Kg/d)	84.7(81.9,90.6)	84.5(80.7,90.2)	84.2(81.2,88.6)	84.5(80.8,90.3)	84.4(79.4,89.1)	84.5(81,90.3)
Energy intakes at discharge (Kcal/Kg/d)	108.3(99.9,116)	111.6(100.5,119.4)	119.2(111.4,122)*^b^*	110.6(100.3,118.4)*^b^*	109.2(100.5,117)	111.2(100.3,119)
Length of hospital stay (d)	19.5(14.5,31.5)	21.5(14.5,34.38)	31.5(25.63,44.5)*^b^*	21(14,32.5)*^b^*	23(17,38.5)	21(14,33.88)
Weight at discharge (g)	2290(2100,2505)*^a^*	2140(2016,2344)*^a^*	2005(1894,2098)*^b^*	2160(2035,2370)*^b^*	2200(2043,2330)	2150(2025,2370)
Changes in WAZ during hospitalization (SD)	−1.05(−1.49,−0.83)	−1.08(−1.35,−0.83)	−1.07(−1.61,−0.93)	−1.08(−1.35,−0.83)	−1.08(−1.39,−0.92)	−1.08(−1.35,−0.83)
Changes in HAZ during hospitalization (SD)	−0.56(−1.15,−0.02)	−0.55(−1.13,−0.05)	−0.47(−1.04,0.34)	−0.55(−1.13,−0.05)	−0.85(−1.6,−0.32)	−0.53(−1.09,−0.03)
Changes in HCAZ during hospitalization (SD)	−0.65(−1.34,−0.08)*^a^*	−0.38(−0.96,0.21)*^a^*	−0.06(−0.64,0.59)	−0.43(−1.01,0.18)	−0.78(−1.45,−0.02)	−0.41(−0.98,0.21)
Changes in WAZ during follow-up (SD)	2.62 ± 1.2*^a^*	1.31 ± 1.13*^a^*	0.92 ± 1.13*^b^*	1.53 ± 1.23*^b^*	0.37 ± 1.16*^c^*	1.59 ± 1.2*^c^*
Changes in HAZ during follow-up (SD)	0.83(−0.32,1.91)	1(0.04,1.86)	−0.76(−1.27,1)*^b^*	1.02(0.05,1.9)*^b^*	1.43(0.78,2.37)*^c^*	0.92(−0.02,1.85)*^c^*

a, a significant difference between infants with or without overweight/obesity; b, a significant difference between infants with or without short stature; c, a significant difference between infants with or without thinness; SGA, small for gestational age; HDP, hypertensive disorders of pregnancy; GDM, gestational diabetes mellitus. BPD, bronchopulmonary dysplasia; ICH, intracranial hemorrhage; NEC, necrotizing enterocolitis; EUGR, extrauterine growth restriction; EN, enteral nutrition; PN, parenteral nutrition; WAZ, weight-for-age Z−score; HAZ, height-for-age Z−score; HC, head circumference; HCAZ, HC-for-age Z−score; BMI, Body Mass Index; BMIZ, BMI-for-age Z-score.

Finally, the results of logistic regression analysis as listed in Table 4 indicated the risk factors of overweight/obesity, short stature, and thinness at age 3−6 years of follow−up were longitudinal EUGR on HC at discharge, cross−sectional EUGR on height at discharge and delayed initiation of EN, respectively. Birth weight, weight at discharge, and changes in WAZ or HAZ during follow-up were also independent impact factors of poor physical growth outcomes.

**TABLE 4:** Multilogistic regression model of overweight/obesity, short stature, and thinness at age 3-6 years.

Variable	*p*	OR	95% CI
Model of overweight/obesity			
Longitudinal EUGR for HC at discharge	0.022	2.193	(1.121-4.291)
Birth weight (g)	0.002	1.001	(1-1.002)
Weight at discharge (g)	0.001	1.002	(1.001-1.003)
Changes in WAZ during follow-up (SD)	<0.001	4.172	(2.986-5.828)
Constant	<0.001	<0.001	
Model of short stature			
Cross-sectional EUGR for height at discharge	<0.001	34.848	(6.819-178.092)
Birth weight (g)	0.001	0.997	(0.995-0.999)
Changes in HAZ during follow-up (SD)	<0.001	0.121	(0.056-0.262)
Constant	0.198	1.162	
Model of thinness			
Days to start EN (days)	0.03	1.178	(1.016-1.399)
Changes in WAZ during follow-up (SD)	<0.001	0.116	(0.065-0.207)
Changes in HAZ during follow-up (SD)	<0.001	4.564	(2.837-7.342)
Constant	<0.001	0.044	

EUGR, extrauterine growth restriction; HC, head circumference; WAZ, weight-for-age Z−score; HAZ, height-for-age Z−score; EN, enteral nutrition.

## Discussion

### Growth pattern of small for gestational age, appropriate for gestational age, and large for gestational age preterm infants

Notably, the changes in *Z*−scores differed significantly among SGA, AGA, and LGA preterm infants. They all encountered obvious declines in *Z*−scores during hospitalization and increased in varying degrees after discharge. Small for gestational age infants had an up-cross of *Z*−scores while LGA infants had a down−cross of *Z*−scores from birth to follow−up. The gap at birth converged toward zero at age 3−6 years. Similar findings were described in a cohort study conducted by Vandana et al. (14), where catch−up growth in SGA and catch−down growth in LGA were regarded as goal−seeking paths that brought infants back to their normal growth channels.

Most of the SGA infants achieved catch−up growth (WAZ or HAZ ≥ −1SD) by the age of 3−6 years, which was noticed as an almost ubiquitous consequence due to their genetic potential in some former studies (15). Small for gestational age infants are predisposed to a large size but limited by the prenatal factors such as HDP and multiple pregnancies. Once the restriction from the intrauterine environment is lifted, their growth potential is stimulated to help them erase the prenatal deficit (14). Besides, seven SGA infants were overweight or obese at follow-up, and even one had a WAZ less than −1 SD at the same time because of the imbalance between height and weight gain, which suggests that the post−discharge growth assessment should focus on the complete nutrition assessment including height, weight, HC, and BMI, rather than one single measurement (16).

Large birth weight and sustained increase in WAZ after discharge led to a high incidence (23.5%) of overweight/obesity in the LGA group. Childhood obesity, mostly due to an excessive calorie intake and a genetic susceptibility to weight gain, add to the risk of cardiometabolic diseases in adulthood such as hypertension, coronary heart disease, and diabetes (17, 18). Therefore, a persistent nutrition monitoring in conjunction with targeted dietary and exercise intervention of premature infants after discharge is essential, especially for the LGA infants (19).

### Nutrition support during hospitalization

The advances in perinatology and neonatology have dramatically reduced mortality in preterm infants, yet increased the number of low birth weight, multiple births, and critically ill newborns in need of mechanical ventilation. However, the data did not show significant increase in the incidences of sepsis, NEC, and EUGR from 2016 to 2018. This can be attributed to the close cooperation between the nutrition support team and multidisciplinary team as well as the improvement we made in nutrition support in accordance with the newly published guidelines (12), including the increase of breastfeeding rate, the early start of EN and PN, and optimal calorie intakes during hospitalization. A number of studies demonstrated that human milk protects against NEC and should be the first choice for feeding the preterm and low-birth-weight newborns (20, 21). The percentage of breastfeeding in this study was relatively low and ranged from 11.8% to 22.2% owing to the shortage of a human milk bank. According to the Chinese Guideline of Nutrition Support for Neonates (11), EN and PN support can be safely administered within 24 h after birth, and the start time of EN was found to influence the occurrence of thinness at follow-up in the regression model. However, the start of both EN and PN were delayed in our study for concern for complications such as feeding intolerance, NEC, and upper gastrointestinal hemorrhage, even though slightly earlier administration of EN was found in 2018. In these aspects, the current nutrition policies still need further amendments to keep up with the medical practices and alleviate the impairment in physical and neural developments (22).

### Relationship between the diagnosis of EUGR and physical growth outcomes

Although the majority of preterm infants had returned to their normal growth paths by the age of 3−6 years, cases of overweight/obesity, short stature, and thinness cannot be ignored. The diagnosis of EUGR at an early stage may be used as detection marker for these unsatisfactory developmental results. Takayanagi et al. demonstrated that in very low birth weight infants, cross-sectional weight EUGR was associated with short stature and thinness around the age of 6 years, whereas obesity was rarely seen in this group (23).

In contrast, the longitudinal EUGR on HC at discharge was identified as an independent risk factor for overweight/obesity in our study. The dysplasia of the nervous system can influence the formation of an unhealthy lifestyle, and thus obesity (24, 25). Among the three growth parameters, the deficit of nutrition first manifests as weight loss, then followed by the stagnant in height and HC growth. The premature infants with longitudinal EUGR on HC might have severe malnutrition, combined with uncertain damage in the nervous system or other undiscovered metabolic diseases, resulting in the uneven growth in weight and height. Further research is needed to elucidate the correlation between EUGR on HC and overweight/obesity.

Within our expectation, cross-sectional EUGR on height at discharge was one of the major predictors for short stature at age 3−6 years. Short stature is closely associated with congenital genetic factors since half of the children with short stature was originated from SGA in our data. Matsumoto et al. (26) revealed that approximately 10% of SGA infants fail to reach normal growth at 2 years old and can be treated with growth hormone, especially for children with low gestational age. Nagasaka et al. (27) also proved that the chances of developing short stature at 3 years old were 4.5−fold higher among SGA infants than non-SGA infants. Several studies have demonstrated that the diagnosis of longitudinal EUGR is more strict and accurate than cross-section EUGR (4, 5). However, SGA infants are often ignored in the longitudinal EUGR since they are born with intrauterine growth restriction and they do not have much room for the continuous decline in HAZ. Compared with the longitudinal EUGR, the diagnosis of the cross−sectional EUGR on height is less rigorous, but more suitable for warning short stature to some extent.

The rates of EUGR on weight, both cross−sectional and longitudinal, were relatively high as a result of physiological weight loss after birth and may not reflect the real state of body growth. Maiocco et al. (28) proposed a new concept of longitudinal “post-loss” EUGR, namely, calculating the change in *Z*−scores from 14−21 days to discharge to avoid the effect of physiological weight loss, and discovered that post-loss EUGR on HC was an independent risk factor for neurodevelopmental impairment. Further research should be conducted to reveal whether “post−loss” EUGR can better predict adverse physical development outcomes.

There are several limitations in this study. This is a retrospective single center study with low follow-up rate. The data obtained by telephone inquiry may be insufficient of consistency and accuracy. Besides, the factors that may affect growth during childhood, such as parental information, nutrition after discharge, and diseases during follow−up, were not included.

## Conclusion

The growth trajectories of preterm newborns tended toward the normal direction. However, it is possible to observe short stature in SGA and obesity or overweight in LGA children. The longitudinal EUGR on head circumference and cross-sectional EUGR on height were associated with overweight/obesity and short stature, respectively. At the same time, the early start of EN may prevent preterm infants from thinness at age 3−6 years. The post-discharge monitoring of growth and the cultivation of a healthy lifestyle played a prominent role in the optimal physical development of preterm infants.

## Data availability statement

The raw data supporting the conclusions of this article will be made available by the authors, without undue reservation.

## Ethics statement

The studies involving human subjects were approved by the Ethics Committee of Shanghai Children’s Medical Center. Written informed consent was obtained from subjects’ parents.

## Author contributions

SL and HF: methodology, formal analysis, data curation, writing the original draft, reviewing, and editing. RZ, GZ, and LP: data curation and investigation. FB: conceptualization. LH: conceptualization, supervision, project administration, and funding acquisition. All authors contributed to the article and approved the submitted version.
